# Selective and Fast Cortisol Sensing in Human Saliva Using Graphene‐Based On‐Chip Field‐Effect Transistors

**DOI:** 10.1002/smsc.70405

**Published:** 2026-09-27

**Authors:** Bajramshahe Shkodra, Ksenija Arslanova, Eva Fellinger, Tanja Stimpel‐Lindner, Sebastian Klenk, Sebastian Hauk, Kathrin C. Knirsch, Andreas Hirsch, Kristiina Iljin, Tarja K. Nevanen, Annette Schmidt, Georg S. Duesberg

**Affiliations:** ^1^ Institute of Physics & Research Center SENS Faculty of Electric Power Systems and Information Technology University of the Bundeswehr Munich Neubiberg Germany; ^2^ Institute for Sports Science Faculty of Human Sciences University of the Bundeswehr Munich Neubiberg Germany; ^3^ Department of Chemistry and Pharmacy Friedrich‐Alexander University of Erlangen‐Nuremberg (FAU) Erlangen Germany; ^4^ VTT Technical Research Centre of Finland Espoo Finland

**Keywords:** biosensing, cortisol, electrolyte‐gated FET, ELISA, GFET, immunosensors, saliva, sweat

## Abstract

Monitoring of biomarkers in body fluids is highly sought after for medical diagnostics and tracking health conditions. Conventional analytical methods require centralized facilities and complex protocols, limiting applicability for real‐time or point‐of‐care use. In recent years, graphene‐based transducers have shown high potential for miniaturized biosensing, however they have so far been hardly tested in real‐case scenarios. In this work, electrolyte‐gated graphene field‐effect transistors (EG‐GFETs) are developed for the detection of the primary stress hormone cortisol in human saliva. The sensor consists of chemical vapor deposition (CVD)‐grown graphene functionalized with perylene bisimide (PBI) linkers for immobilizing anticortisol (17) Fab fragments. Cortisol is detected over a dynamic range from 100 fM to 1 μM, covering physiologically relevant concentration ranges. The measurements show high correlation with enzyme‐linked immunosorbent assay (ELISA) tests performed in parallel on the same samples. The immediate, label‐free sensing of biomarkers in human saliva represents a major advance in miniaturized biosensing and can potentially be extended to further analytes and integrated portable monitoring units.

## Graphical abstract

Electrolyte‐gated graphene field‐effect transistors (EG‐GFETs) functionalized with anticortisol Fab fragments enable rapid, label‐free detection of cortisol in human saliva within 10 min. Sensitive operation in physiologically relevant media, together with direct validation against ELISA, demonstrates the potential of this platform for future portable and real‐time stress monitoring.

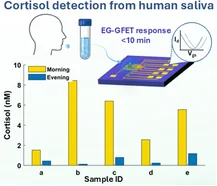

## 1 Introduction

Detection of biomarkers has become essential to modern healthcare, medical diagnostics, performance diagnostics, and early disease detection. Nanomaterial‐based biosensors have shown great potential for this, particularly for the quantification and continuous monitoring of biochemical markers in body fluids. They potentially allow the detection of various analytes, such as glucose, lactate, disease biomarkers, and hormones, with high sensitivity and selectivity [1, 2]. Among these, the human stress hormone cortisol is of particular interest as it plays a central role in the body’s endocrine response to stress. Cortisol production is tightly regulated by the hypothalamic–pituitary‍–‍adrenal (HPA) axis (Figure 1a). Upon exposure to a sufficiently strong stressor, the hypothalamus secretes corticotropin‐releasing hormone (CRH), which stimulates the pituitary gland to release adrenocorticotropic hormone (ACTH). ACTH subsequently triggers the adrenal cortex to produce glucocorticoids, primarily cortisol in humans. Cortisol is then released into the bloodstream and, through passive diffusion, is present in multiple other biofluids, including sweat, saliva, urine, and interstitial fluid [3].

**FIGURE 1:**
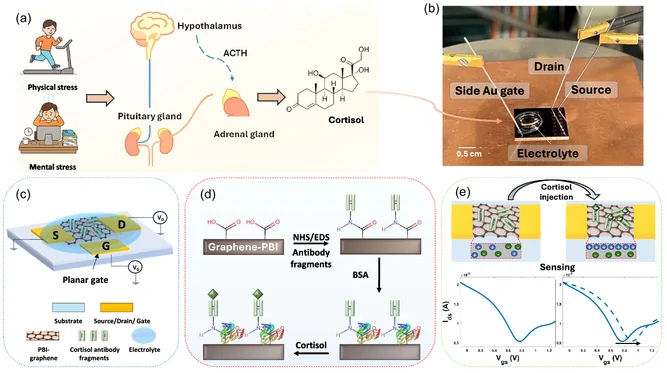
(a) Schematic of cortisol secretion triggered by stress stimulation. (b) Photograph of electrolyte‐gated graphene field‐effect transistors (EG‐GFETs) during measurement. (c) Schematic representation of an EG‐GFET with a functionalized graphene channel contacted by source and drain electrodes. (d) Process flow of the functionalization of the EG‐GFET channel via covalent binding of cortisol Fab antibody fragments onto perylene bisimide (PBI). (e) Schematic representation of the detection mechanism showing a shift of the Dirac voltage (*V*_Dirac_) after binding of the analyte (dashed curve).

In addition to this endocrine cascade, cortisol secretion follows a well‐established circadian rhythm, with concentrations peaking in the early morning and reaching their lowest levels in the evening [4]. Beyond circadian variation, cortisol also exhibits event‐related dynamics in response to acute stressors. Capturing this acute component is particularly important because acute stress can significantly affect human cognition and operational performance. Experimental studies show that acute stress impairs decision quality, with deficits becoming especially pronounced under time pressure conditions that frequently characterize operational environments [5, 6].

Real‐time stress monitoring in mission‐critical contexts, e.g., emergency medicine, response in catastrophic scenarios, or critical operations, could therefore be highly valuable, as maintaining operational readiness is essential and decision errors under stress can have detrimental consequences for team safety and mission outcomes. Because acute stress is transient and cortisol secretion is time‐shifted, typically peaking 20–30 min after stressor onset, single time‐point measurements are insufficient, relevant peaks may be missed, and the intensity or timing of acute stress responses may be mischaracterized [7, 8]. Repeated measurements therefore improve interpretability by capturing the trajectory of the response, separating acute reactivity from the circadian background, and enabling objective monitoring in field conditions where stressors occur unpredictably.

Although clinical diagnostic methods, such as electrochemiluminescence, radioimmunoassays, and enzyme‐linked immunosorbent assays (ELISA), provide reliable cortisol quantification, they typically require complex instrumentation, time‐consuming assay procedures, and trained personnel, limiting their applicability outside centralized laboratory settings [9, 10]. Consequently, there is a growing demand for diagnostic approaches that enable rapid, accessible, and continuous cortisol monitoring. To address this need, recent research has increasingly focused on novel biosensing technologies, including electrochemical biosensors, optical sensors, surface plasmon resonance (SPR), and electrolyte‐gated field‐effect transistors (EG‐FETs) [11, 12, 13, 14, 15, 16, 17].

Among these, EG‐FETs have garnered particular interest due to their inherent signal amplification, low detection limits, and compatibility with label‐free sensing, enabling highly sensitive cortisol detection [9, 16, 18, 19, 20, 21, 22]. In particular, graphene‐based EG‐FETs (electrolyte‐gated graphene field‐effect transistors (EG‐GFETs)) have demonstrated exceptional potential owing to graphene’s high electron mobility and tunable Fermi level, which together enhance charge–transfer efficiency at the sensing interface [9, 23, 24, 25]. Moreover, graphene’s atomically thin structure ensures that charge carrier modulation induced by biomolecular interactions occurs in close proximity to the conductive channel, thereby maximizing transduction sensitivity. Its large specific surface area and versatile surface chemistry further enable efficient functionalization with biorecognition elements such as antibodies, antibody fragments, or aptamers, facilitating selective cortisol detection. These attributes allow EG‐GFETs to operate at low gate voltages, rendering them highly attractive for wearable and point‐of‐care cortisol monitoring applications.

Building on these advantages, EG‐GFETs have been actively explored for cortisol detection, predominantly in buffer solutions or artificial biological fluids. Gilani et al. reported an EG‐GFET capable of detecting cortisol in 1× PBS (phosphate‐buffered saline) with a limit of detection (LOD) of 1 pM [26], while Ku et al. achieved an LOD of 10 pg/mL in artificial tears [21]. More recently, Kim et al. developed an EG‐GFET functionalized with an oligo(phenylene‐ethynylene)amine linker, enabling cortisol detection down to 500 fM in artificial sweat [9]. Despite these promising results, the evaluation of EG‐GFETs has largely been confined to buffer solutions, i.e., artificial media, and to date comparative benchmarking against state‐of‐the‐art analytical techniques in real biological fluids has not been reported.

Here, we present an EG‐GFET that enables a fast, label‐free, and quantitative cortisol detection in human saliva (Figure 1b). To achieve this, the graphene transducer is functionalized with perylene molecules serving as linker units for the immobilization of anticortisol (17) Fab biorecognition elements (Figure 1c).

Perylene molecules have been extensively investigated due to their excellent optical and electronic properties, as well as their strong self‐assembled monolayer behavior [27, 28, 29]. In particular, perylene‐3,4:9,10‐tetracarboxylic diimides and their N,Nʹ‐‍substituted derivatives, commonly referred to as perylene bisimides (PBIs, Figure S1), are high‐performance n‐type organic semiconductors exhibiting exceptional chemical, thermal, and photostability [30]. In this work, the perylene molecules not only enable covalent immobilization of the anticortisol (17) Fab (Figure 1d) but also act as a physical barrier that protects the graphene channel from ambient impurities. This protection is achieved through the functional layer‐assisted transfer (FLaT) method developed in our laboratory [29].

Our previous work established the FLaT approach as a reproducible strategy for noncovalent graphene functionalization using PBI linker molecules, enabling stable and chemically addressable sensing interfaces. Building upon this strategy, the present study extends its application to EG‐GFET biosensors for selective cortisol detection in physiologically relevant media. In particular, the demonstrated operation in human saliva, together with direct benchmarking against ELISA measurements, represents an important step toward the translation of this strategy into practical biosensing applications.

Successful surface functionalization was confirmed by comprehensive characterization of the layer‐by‐layer modification of chemical vapor deposition (CVD)‐grown graphene with PBI and subsequent Fab immobilization using Raman spectroscopy, X‐ray photoelectron spectroscopy (XPS), and electrochemical measurements. The integrity and functionality of the sensing interface were further validated through the electrical characterization of the EG‐GFETs.

The Fab–PBI–functionalized EG‐GFET platform enables sensitive cortisol detection over a dynamic range spanning 100 fM–1 μM within less than 10 min. Cortisol binding induces a reorganization of interfacial charges and counterions within the immobilized Fab layer, resulting in effective electrostatic gating of the graphene channel and a positive shift of *V*_Dirac_ (the gate potential at which the channel reaches minimum conductivity) toward higher gate voltages (*V*_GS_) (Figure 1e). This measurable shift in *V*_Dirac_ serves as a direct electrical output signal. Such an electrically transduced response provides a pathway toward future integration into compact and portable electronic sensing platforms. Furthermore, robust sensing performance was demonstrated in 1× PBS, artificial sweat, and human saliva, covering the physiological cortisol concentration range of 100 pM–100 nM [16].

Most importantly, the measurements in human saliva showed a high correlation with those obtained using a conventional ELISA test. For this purpose, a series of ten samples from five probands was analyzed anonymously using both methods. This validation of the EG‐GFET performance with human fluids against established techniques is rarely demonstrated and establishes the technology as a promising platform for biosensing. Furthermore, the fast detection capability could enable real‐time monitoring outside conventional laboratory settings through integration into compact electronic readout units.

## 2 Results and Discussions

The EG‐GFETs employed in this study were fabricated from CVD‐grown graphene, serving as the conductive channel. The fabrication process is fully described in the Experimental Section and schematically presented in Figure S2.

### 2.1 PBI‐Graphene Characterization

In‐house CVD‐grown graphene was analyzed by Raman spectroscopy. A typical Raman spectrum of graphene is shown in Figure 2a (blue). Two main peaks are clearly observed: the prominent 2D band located at 2680 cm^−1^ and the G band centered at 1588 cm^−1^. A weak D band (1345 cm^−1^), which corresponds to the defective non sp^2^ bonds in graphene, is also observed. The symmetric and narrow 2D band had a full width at half maximum (FWHM) of about 19.16 cm^−1^. The high 2D to G peak intensity ratio (*I*_2D_/*I*_G_ = 2.3), the single‐component narrow 2D peak, and the very low defect density (*I*_D_/*I*_G_ = 0.04) evidence the high crystalline quality of monolayer graphene [31].

**FIGURE 2:**
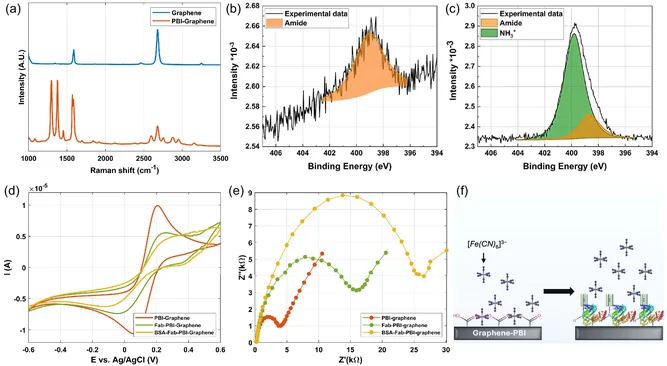
(a) Raman spectra of as‐transferred graphene (blue) and PBI‐graphene (orange), showing graphene and PBI‐related peaks. (b) X‐ray photoelectron spectroscopy (XPS) spectra of the N 1s peak intensities for PBI‐graphene and (c) Fab‐functionalized PBI‐graphene. (d) Cyclic voltammetry (CV) measurements demonstrating the change in peak current with increasing modification complexity. (e) Electrochemical impedance spectroscopy (EIS) showing changes in the charge–transfer resistance (*R*_CT_) of the redox reporter ferricyanide ion ([Fe(CN)_6_]^3^^−^) with increasing modification complexity. (f) Schematic representation of the diffusion of [Fe(CN)_6_]^3^^−^ toward the functionalized PBI‐graphene surface.

The Raman spectrum of PBI‐functionalized graphene shown in Figure 2a (orange) displays distinct features characteristic of the PBI derivative [29]. Peaks observed around 1297 cm^−1^ correspond to vibrational modes of the PBI core, consistent with previous reports that assign similar modes to the perylene moiety on graphene surfaces [28]. Additional peaks at higher wavenumbers arise from terminal functional groups such as carboxylic acid moieties, as depicted in the molecular structure (Figure S1). The intensity ratio between the PBI‐related peak at 1297 cm^−1^ and the graphene G peak (*I*_1297_/*I*_*G*_ = 1.2) indicates a densely packed molecular layer, supporting the formation of a uniform and well‐ordered functionalization layer. This ratio has been established in the literature as a reliable metric to assess molecular packing density on graphene, correlating with efficient surface coverage [28]. These findings confirm the effectiveness of the FLaT method for producing stable, high‐quality PBI‐functionalized graphene interfaces.

### 2.2 Biofunctionalization Validation

Following the successful functionalization of graphene with PBI, biofunctionalization with Fab fragments was subsequently performed. The sequential deposition of Fab fragments onto the PBI‐graphene surface was confirmed by monitoring the evolution of characteristic elemental peaks in the XPS spectra. Both survey spectra (see Figure S3) and high‐resolution N 1s spectra were acquired. The N 1s region exhibits a clear increase in intensity after Fab deposition.

The PBI molecule contains two nitrogen species that exhibit nearly identical N 1s binding energies due to their similar chemical environments [28]. Consequently, the N 1s spectrum of the PBI‐only sample is dominated by a single, slightly broadened component, whose relatively low intensity is consistent with monolayer coverage (see Figure 2b). In contrast, the Fab‐functionalized sample exhibits a pronounced increase in total nitrogen content, arising from nitrogen‐containing groups within the Fab fragments, indicating their presence at the PBI‐graphene interface (see Figure 2c). Moreover, an additional component emerges at a higher binding energy (399.8 eV), indicative of protonated amine species introduced upon Fab immobilization [32, 33].

The results obtained with XPS are further corroborated by electrochemical measurements (see the setup in Figure S4a). Electrochemical validation of the Fab fragment immobilization and the BSA blocking layer was performed by monitoring the evolution of the oxidation/reduction peak currents during cyclic voltammetry (CV) and by examining changes in the Nyquist plots of the electrochemical impedance spectroscopy (EIS) response, with particular attention to the charge–transfer resistance (*R*_CT_), inferred from the diameter of the semicircle [34].

Successful functionalization is evidenced by the cyclic voltammograms shown in Figure 2d. The bare PBI‐graphene active layer exhibits the highest reduction peak current, approximately 10 μA. Following the successive biofunctionalization steps, a progressive decrease in the peak current is observed. This decrease reflects the increasing hindrance to the diffusion of the redox reporter [Fe(CN)_6_]^3^^−^ toward the active layer, caused by the Fab fragment grafting and by the BSA blocking layer, which effectively act as insulating barriers [35].

Figure 2e displays the Nyquist plots obtained from EIS measurements and fitted to a Randles circuit [36]. The bare PBI‐graphene layer exhibits a small semicircle corresponding to a low *R*_CT_ of 3.68 kΩ, indicative of facile charge–transfer kinetics. Upon immobilization of the Fab fragments, the semicircle diameter increases noticeably, resulting in an *R*_CT_ value of 15.9 kΩ, consistent with restricted access of [Fe(CN)_6_]^3^^−^ to the electrode surface due to the insulating biomolecular layer. Subsequent modification with BSA further increases the *R*_CT_ to 26.5 kΩ, confirming the formation of an additional blocking layer on the active surface [35, 37]. In addition, Bode plots of impedance and phase angle (see Figure S4b) were acquired to further evaluate changes at the PBI‐graphene surface during biofunctionalization. A clear increase in impedance is observed following biofunctionalization, accompanied by a decrease in the phase angle (from −54.06° for bare PBI‐graphene to −60.95° after biofunctionalization), indicating a more resistive interfacial environment. These changes provide additional evidence of successful biofunctionalization of the graphene channel [37, 38]. A schematic representation illustrating the progressively restricted diffusion of [Fe(CN)_6_]^3^^−^ toward the PBI‐graphene surface upon biofunctionalization is shown in Figure 2f.

### 2.3 EG‐GFET Biosensing Operation

In an EG‐GFET, the charge‐carrier density in the graphene channel is modulated by sweeping the potential applied to the gate electrode immersed in the electrolyte. At low gate voltages (left side, p‐branch), holes dominate the conduction, whereas at higher gate voltages (right side, n‐branch), electrons become the majority carriers, resembling an ambipolar transistor (behavior). These two regimes are separated by the Dirac point (*V*_Dirac_), which corresponds to the potential of minimal conductivity, also referred to as the charge neutrality point. Monitoring shifts in *V*_Dirac_ allows detection of changes in the graphene chemical environment, thereby enabling the electrical readout of biochemical interactions [39].

The typical ambipolar transfer characteristic of the fully biofunctionalized EG‐GFET device is shown in Figure S5a, exhibiting a *V*_Dirac_ value of 0.76 V and a calculated mobility of 6.94 cm^2^/Vs, measured in 1× PBS. This is considerably lower than what we have previously reported for CVD‐grown graphene‐based back‐gated devices [40] and CVD‐grown graphene‐based biosensors [41]. The discrepancy reflects differences in capacitance considerations and measurement conditions: back‐gated devices account for the gate oxide capacitance, while the previously reported biosensors were operated in low‐ionic‐strength electrolytes.

Therefore, it is important to note that mobility is calculated for fully functionalized EG‐GFET devices following the method of Soikkeli et al. [42], which explicitly accounts for the ionic strength of the electrolyte. In this case, the elevated ionic strength of 1× PBS (0.156 M) significantly reduces the capacitance of the electrical double layer, resulting in an apparent reduction of the calculated mobility.

While carrier mobility is an important parameter reflecting graphene quality, it should be noted that there is no direct correlation between mobility and the magnitude of the Dirac point shift upon analyte binding [41].

The gate current (*I*_G_), i.e., leakage current, is an important factor to consider in electrolyte‐gated configurations. This effect is typically absent in traditional back‐gated or dielectric‐gated FETs but may occur in EG‐FETs due to possible electrochemical reactions [43]. Such leakage current is unwanted in any transistor operation because it leads to energy loss, compromises device performance, and introduces noise to the signal of interest. For these reasons, to ensure that the measured signal arises from the cortisol–Fab interaction rather than secondary reactions, *I*_G_ was monitored at every measurement stage. The EG‐GFET devices exhibit at least two orders of magnitude lower *I*_G_ compared to drain current (*I*_DS_), demonstrating good device behavior (Figure S5a).

Complementing the low leakage current, the linear relationship between *I*_DS_ and drain‐source voltage (*V*_DS_) confirms ohmic contact between graphene and the metal electrodes (see Figure S5b). This indicates that the Schottky barrier and contact resistance at the source and drain have minimal impact [44]. Additionally, the effective modulation of the source‐drain current by *V*_GS_ demonstrates proper field‐effect control of the channel conductivity, which is essential for reliable device operation and sensing performance.

In summary, surface characterization by Raman spectroscopy, XPS, and electrochemical measurements confirmed the controlled immobilization of cortisol Fab fragments onto the PBI‐graphene transducer, enabling an electrical readout. Thus, our process flow ensures reliable EG‐GFET operation for the biosensing of targeted analytes.

Because sensing performance critically depends on the accessibility of the immobilized recognition elements, the orientation of the Fab fragments is also an important consideration. Since Fab immobilization is achieved through EDC/NHS coupling, a random orientation of the Fab fragments is expected as carbodiimide coupling generally occurs through accessible amine groups distributed across the protein surface rather than through site‐specific attachment [45]. Although the orientation of the immobilized Fab fragments was not investigated in the present study, the observed sensitivity and selective cortisol detection indicate that a sufficient fraction of the immobilized Fab fragments retains accessible antigen‐binding sites, enabling efficient cortisol recognition and electrical transduction.

As shown in Figure 3a exposure of the Fab‐functionalized EG‐GFET biosensors to cortisol results in concentration‐dependent shifts in *V*_Dirac_. The binding of cortisol to the immobilized Fab fragments modifies the interfacial electrostatic environment at the graphene–electrolyte interface. This perturbation alters the electrical double layer and leads to a capacitive modulation of the carrier density in the graphene channel. Consequently, a higher positive gate voltage is required to restore charge neutrality, giving rise to the observed positive shift in *V*_Dirac_. The Fab‐functionalized EG‐GFET biosensors detect cortisol from 1 pM to 1 μM in undiluted 1× PBS, fully covering the physiologically relevant concentration range highlighted in green Figure 3b.

**FIGURE 3:**
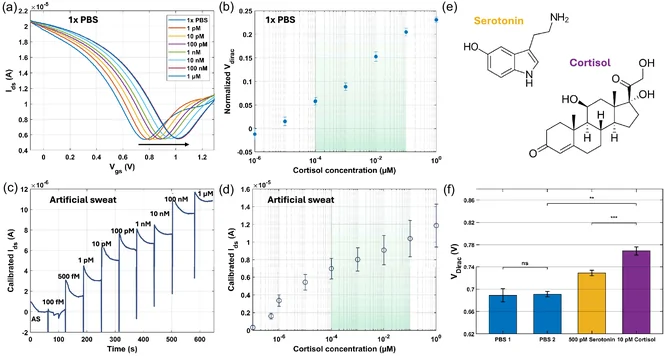
(a) Transfer curve shifts of Fab‐functionalized EG‐GFETs upon exposure to varying cortisol concentrations. (b) Normalized Dirac point shifts vs. cortisol concentration (1 pM–1 μM). (c) Real‐time measurements and exposure of Fab–functionalized EG‐GFETs to cortisol in undiluted artificial sweat led to concentration‐dependent increases in the drain‐source current (*I*_DS_). (d) Normalized *I*_DS_ vs. cortisol concentration (100 fM–1 μM). The green boxes in (b) and (d) highlight the physiologically relevant cortisol concentration range. Error bars in (b) and (d) represent the standard error of the mean (*N* = 8 devices per concentration). (e) Chemical structure of serotonin and cortisol. (f) Selectivity evaluation of the cortisol EG‐GFET biosensor. PBS 2 represents PBS reinjection. Error bars represent SEM (*N* = 5). **p ≤ 0.01, ***p ≤ 0.001.

The ability of the EG‐GFET to operate effectively in a high‐ionic‐strength environment is facilitated by the compact structure of the Fab fragments. As described in the structural analysis by Eronen et al. [46], the light‐chain complementary determining region (CDR) loops of the (17) Fab belong to the L1‐10–2, L2‐8–1, and L3‐7–1 clusters, representing the shortest loop lengths within their respective families, while the heavy‐chain loops fall into the H1‐13–1, H2‐10–2, and H3‐12–1 clusters, corresponding to near‐average lengths. Because no elongated or protruding CDR loops are present, the antigen‐binding site adopts a relatively flat geometry with a shallow cavity that accommodates the hapten (cortisol). Although the overall Fab fragment dimensions (approximately 7 × 4 × 3 nm) exceed the Debye length in 1× PBS, this compact binding‐site architecture places the cortisol‐binding pocket closer to the graphene surface than would be expected for conventional full‐length antibodies. Furthermore, the sensing mechanism is not solely attributed to direct electrostatic interaction of cortisol with graphene but also to the redistribution of charges and counterions within the immobilized Fab layer upon cortisol binding, resulting in modulation of the electrical double layer and the graphene channel conductivity.

The performance of the Fab‐functionalized EG‐GFET was further evaluated through real‐time monitoring in 1× PBS. Upon injection of cortisol at concentrations ranging from 1 pM to 1 μM, the calibrated *I*_DS_ exhibited a concentration‐dependent increase as shown in Figure S6. The calculated LOD was determined to be 30 fM, highlighting the sensor’s capability for ultrasensitive cortisol detection in physiologically relevant fluids.

Having demonstrated robust performance in 1× PBS, the device was further challenged in more physiologically relevant environments, such as artificial sweat (see the composition in Table S1), to evaluate the sensor’s sensitivity under more realistic conditions. Additionally, the cortisol concentration range was extended to lower levels, from 100 fM up to 1 μM (see Figure 3c). The device successfully detected cortisol across the entire tested concentration range in artificial sweat, as shown in Figure 3d, demonstrating reliable sensitivity under complex matrix conditions.

To verify the selectivity of the detection, the EG‐GFET biosensor was exposed to potential interfering analytes, such as serotonin (see Figure 3e). Serotonin was specifically chosen due to its relevance as a biomarker for mental health disorders. The sensor response was evaluated by injecting serotonin at concentrations 50 times higher than that of cortisol. A negligible response to the interfering analyte was observed, whereas the EG‐GFET biosensor exhibited a clear and specific response to cortisol at concentrations as low as 10 pM, as shown in Figures 3f and S7.

### 2.4 Cortisol Quantification and Cross‐Validation in Circadian Cycles

For cross‐validation of EG‐GFET performance, cortisol ELISA tests were carried out using identical saliva samples. Numerous studies have demonstrated a strong correlation between cortisol levels and the circadian rhythm, with elevated concentrations observed in the morning, decreasing throughout the day, and reaching their lowest levels in the evening [10, 16]. In this study, saliva samples were collected from five participants at different time intervals. The collected saliva was then pipetted on the EG‐GFET, and the cortisol response was obtained within 7 min (Figure 4a). The EG‐GFET immunosensor results for cortisol detection in saliva from the five participants are presented in Figure 4b, while the ELISA results are given in Figure 4c. The low cortisol concentrations measured using the EG‐GFET sensor showed good agreement with those obtained by the standard ELISA technique. For example, in sample **c**, the cortisol concentration determined by EG‐GFET was 0.79 nM, closely matching the ELISA result of 0.80 nM, corresponding to a percent difference of only 1.54%.

**FIGURE 4:**
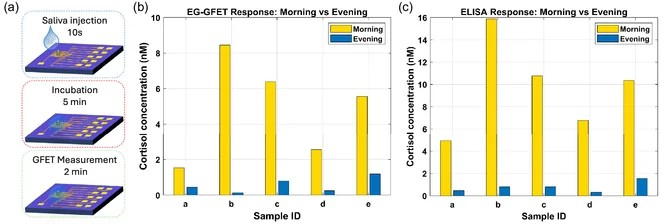
(a) Schematic presentation of the protocol for saliva analysis: following saliva injection, a 5 min incubation period allows interaction with the Fab‐functionalized EG‐GFET sensor, followed by a 2 min measurement. (b) Cortisol detection responses from five participants measured by the EG‐GFET biosensor. (c) Corresponding cortisol concentrations measured by ELISA for the same samples.

At higher cortisol concentrations (morning values), a lower agreement between the two methods was observed. The EG‐GFET results were approximately half of those obtained with the ELISA test. This can tentatively be attributed to the calibration range used for the sensor and to potential sensor saturation in such a complex biological environment. The calibration curve for real saliva samples was established using the exact same concentration points as those employed in the ELISA calibration (0.14–8.83 nM), enabling a direct one‐to‐one comparison between the two methods. Low cortisol values, which fall well within this range, showed a strong agreement between the two methods. However, higher cortisol concentrations approached or slightly exceeded the upper limit of the calibration range (*e.g.*, 6.39 nM measured by EG‐GFET versus 10.77 nM by ELISA). At these higher concentrations, the EG‐GFET sensor response may become nonlinear, leading to an underestimation of cortisol levels. This limitation highlights the importance of extending the calibration curve to cover the full expected physiological range in order to achieve more accurate quantification across all sample conditions.

These results underscore the necessity of rigorous calibration and direct validation against established methods such as ELISA for reliable cortisol quantification in complex biological matrices. Notably, high performance is maintained in 1× PBS, artificial sweat, and real human saliva, demonstrating robust sensing under conditions that closely approximate native biological environments. This study provides the first direct benchmarking of EG‐GFET cortisol‐sensing results against conventional ELISA measurements. Building upon this validation, it is crucial to benchmark the analytical performance of the developed EG‐GFET sensor against recently reported carbon nanomaterial‐based cortisol biosensors. A comparison of these devices is summarized in Table S2, highlighting the unique advantages of the present Fab‐functionalized graphene EG‐GFET in terms of sensitivity, dynamic range, and robustness under physiologically relevant conditions.

## 3 Conclusion

We have demonstrated a label‐free EG‐GFET biosensor functionalized with PBI linkers and anticortisol Fab fragments for sensitive and selective cortisol detection in physiologically relevant body fluids. Comprehensive surface and electrical characterization has confirmed the successful fabrication and reliable transistor operation. The sensor exhibits an ultralow LOD of 30 fM, a broad dynamic range spanning from 100 fM to 1 μM, and stable performance in high ionic strength media, including 1× PBS, artificial sweat, and human saliva. The compact Fab fragment binding geometry promotes efficient charge–transfer signal transduction, enabling robust sensing under biologically relevant conditions.

Cross‐validation against ELISA assays has shown strong agreement at low cortisol concentrations, while deviations at higher levels likely reflect calibration range limitations and indicate opportunities for further optimization. To our knowledge, this study provides the first direct benchmarking of EG‐GFET‐based cortisol sensing against ELISA, underscoring its potential for rapid point‐of‐care stress monitoring and clinical diagnostics.

The rapid sensing response (<10 min) further supports the platform’s suitability for time‐sensitive applications. Upon integration into compact electronic readout systems, this capability could enable real‐time, out‐of‐lab monitoring. Future work will focus on extending the selectivity assessment to structurally related steroid hormones and on multiplexed detection of additional stress‐related biomarkers (e.g., alpha‐amylase), further advancing the platform toward comprehensive stress monitoring.

## 4 Experimental Section

### 4.1 Graphene Growth and Functionalization

Graphene was synthesized on 25 μm thick copper foils (Alfa Aesar, 99.8%, Merck) via CVD. Prior to growth, the copper substrates were treated with oxygen/argon to remove intrinsic carbon contaminants. Graphene growth was carried out at 1060 °C in the central zone of a CVD furnace using a hydrogen‐methane‐argon gas mixture [29]. Immediately after CVD, the Cu‐supported graphene was functionalized by immersion in an aqueous 0.1 mM solution of PBI [47] for 15 min using the FLaT approach [29]. This immediate functionalization serves two critical purposes: (1) it creates a physical barrier that protects the graphene surface from airborne contamination and (2) it promotes the self‐assembly of PBI molecules, forming a stable, noncovalent interface rich in carboxylic groups. This interface is essential for the subsequent immobilization of biorecognition elements, enabling effective biofunctionalization.

### 4.2 Device Fabrication

Graphene was transferred onto Si/SiO_2_ (300 nm oxide thickness) substrates using a PMMA‐assisted wet‐etching process, followed by the removal of the copper foil and PMMA support. Devices were fabricated using two photolithography steps: the first defined the graphene channel (200 μm × 800 μm) and removed excess PBI‐functionalized graphene by oxygen plasma etching, while the second formed Ni/Au source, drain, and gate contacts by thermal evaporation and lift‐off [48]. Finally, residual polymers were removed by extended acetone cleaning and high‐temperature vacuum annealing to ensure clean, reliable transistor operation (see the Supporting Information in Figure S2 for the full procedure).

### 4.3 Material Characterization

The quality of the graphene and PBI‐functionalized graphene channels was characterized using optical microscopy (Axio Imager, Carl Zeiss Microscopy GmbH, Germany), Raman spectroscopy (WITec alpha300 R), atomic force microscopy (AFM, Jupiter XR, Asylum Research–Oxford Instruments), scanning electron microscopy (SEM, ZEISS Crossbeam 550), and XPS (PHI VersaProbeIII). Raman spectra were acquired using a 532 nm laser with a power of 1 mW. XPS measurements were carried out using a microfocused monochromatic Al K*α* source (1486.6 eV) with dual‐beam charge neutralization.

### 4.4 Biofunctionalization

To functionalize the PBI‐graphene channels with antibody fragments, the surface was first activated using carbodiimide coupling chemistry. A 1:1 mixture of 20 mM EDC (1‐ethyl‐3‐(3‐dimethylaminopropyl) carbodiimide hydrochloride) and 50 mM NHS (N‐hydroxysuccinimide) was prepared in MES buffer (2‐(N‍‐‍morpholino)ethanesulfonic acid, pH 6) and drop‐cast onto the PBI‐graphene channels. The devices were incubated for 30 min at room temperature to activate the carboxyl groups of the PBI linker. Given the very low defect density of the CVD graphene (*I*_D_/*I*_G_ = 0.04), EDC/NHS activation is expected to occur predominantly through the carboxyl groups provided by the PBI linker molecules rather than through intrinsic graphene defect sites [29].

After activation, anticortisol (17) Fab antibodies (10 μg mL^−1^ in 1× PBS) were applied to the channels and incubated for 1 h at room temperature to allow covalent attachment. Excess nonbound antibodies were removed by rinsing the devices twice with 1× PBS.

To minimize nonspecific adsorption during sensing measurements, the remaining unreacted PBI‐graphene surface was passivated by applying 50 μL of 1% (w/v) BSA in 1× PBS. The devices were incubated for 30 min at room temperature, followed by two rinsing steps with 1× PBS to remove excess BSA. The devices were used immediately for electrical measurements after the blocking step.

### 4.5 Biofunctionalization Validation

XPS was employed to investigate the successful biofunctionalization. The distinction between PBI‐functionalized graphene and Fab‐functionalized graphene was assessed through analysis of the N 1s XPS spectra, focusing on nitrogen content and its associated chemical bonding states. In addition, the presence or absence of additional impurities was confirmed by measuring a survey spectrum ranging from 0 to 1100 eV. The spectra were fitted using the software CasaXPS. All spectra were shifted to the silicon oxide peak of the biosensor substrate (Si2p, 103.3 eV).

To confirm successful biofunctionalization, CV was performed in 2 mM ([Fe(CN)_6_]^3^^−^) in 0.1 M KCl within a potential range from −0.6 to +0.6 V at a scan rate of 100 mV/s for all the functionalization steps. To further confirm successful biofunctionalization, EIS was first performed in 2 mM of ferricyanide ([Fe(CN)_6_]^3^^−^) in 0.1 M KCl [49] in a frequency range from 0.1 Hz to 100 kHz for all the functionalization steps. The Nyquist plots were then fitted to the Randles circuit.

For both measurements, the PBI‐graphene channel served as the working electrode (with source and drain electrodes short‐circuited to form a single conductive entity), a platinum wire as the counter electrode, and a Ag/AgCl electrode as the reference electrode (see Figure S4). The measurements were performed using a potentiostat (Gamry Reference 3000).

### 4.6 Biosensing

Electrical characterization of the devices was performed using a probe station connected to source measure units controlled via LabVIEW software (National Instruments, USA). To ensure complete coverage of the three metallic electrodes (source, drain, and gate), 70 μL of 1× PBS (pH 7.4) or artificial sweat was applied as the electrolyte [36, 50]. Composition of the 1× PBS and the artificial sweat are given in the Table S1 in the Supporting Information. For saliva‐sensing experiments, 50 μL of human saliva was incubated on the device for 5 min, followed by rinsing with 1× PBS. Final measurements were then conducted in the presence of 70 μL of a 1× PBS electrolyte.

The output characteristics were recorded by varying the *V*_DS_ from −0.02 to 0.02 V for different values of *V*_GS_ (from −0.02 to 0.5 V, with 0.1 V steps). The transfer characteristics were recorded by sweep the *V*_GS_ from −0.2 to 1.3 V, while maintaining the *V*_DS_ constant at 0.01 V. From the transfer characteristic, mobility was extracted using the following formulas:

(1) $$\mu =\frac{1}{C_{\mathrm{EDL}}}\frac{L}{W}\frac{g_{m}}{V_{\mathrm{DS}}},C_{\mathrm{EDL}}=\frac{\varepsilon_{r}\varepsilon_{0}}{\lambda }$$

where *C*_EDL_ is capacitance per unit area of the electric double layer (EDL), *L* is the channel length, *W* is the channel width, *g*_*m*_ is the transconductance, *V*_DS_ is the drain to source voltage, *ε* *_r_* is the relative permittivity of the electrolyte, *ε*_0_ is the permittivity of free space, and *λ* the Debye length.

For each device, ten consecutive transfer characteristics were recorded at baseline (only electrolyte) and at each cortisol concentration. *V*_Dirac_ was extracted by averaging the last three measurements for each device at each concentration, and the overall standard deviation was calculated from measurements across multiple devices. *V*_Dirac_ was calculated according to the following formula:

(2) $$V_{\mathrm{Dirac}}=\frac{V_{D(C)}-V_{D,0}}{V_{D,0}}$$

where *V*_D(C)_ is the Dirac point measured at a given cortisol concentration, and *V*_D, 0_ is the baseline Dirac point prior to cortisol exposure.

For the real‐time measurements, the *I*_DS_ was continuously monitored while the bias voltages were held constant at *V*_DS_ = 0.01 V and *V*_GS_ = 0.4 V. For each device, *I*_DS_ was first recorded under baseline conditions (electrolyte only) for 60 s, followed by measurements at each cortisol concentration. The response was determined by averaging the final 30 s of the *I*_DS_ for each concentration. The overall standard deviation was obtained from measurements collected across multiple devices. During real‐time measurements, the *I*_DS_ showed a linear decrease in absolute value, as shown in Figure S6 (i.e., for this specific device, a decrease of 14.7 nA/s with a coefficient of determination of 97.07%). It was hence possible to subtract this linear trend from the response of the devices at different concentrations of cortisol (calibrated response) [51, 52].

The LOD was calculated according to the following formula:

(3) $$\mathrm{LOD}=\frac{3.3\sigma_{0}}{S}$$

where *σ*_0_ is the standard deviation of the baseline signal and *S* is the sensitivity, defined as the slope of the linear calibration graph at low analyte concentrations [53].

### 4.7 ELISA Measurements—Saliva Sampling and Analysis

Saliva samples were collected via salivettes (Sarstedt, Nümbrecht, Germany). Informed consent was obtained, after which participants were directed to hold the Salivette in their mouth for a minimum of 1 min, moving it side to side without chewing on it. Saliva samples were stored at −20 °C after collection. The analysis was conducted in the in‐house laboratory of UniBw M. Before analysis, the salivettes were thawed at room temperature and centrifuged (Medifuge, Thermo Fisher Scientific, Waltham, MA, USA) at 3000 × g for 15 min immediately prior to testing. Salivary cortisol concentrations were determined using a Human Cortisol Competitive ELISA Kit (Invitrogen, Thermo Fisher Scientific, Waltham, MA, USA). For analysis, the ELISA plate was placed in an Absorbance Microplate Reader (Magellan, Tecan Group Ltd., Switzerland). The optical density was measured at the recommended wavelength of 450 nm. Cortisol concentrations were calculated by interpolation from the standard curve generated for each plate. According to the manufacturer’s specifications, the assay’s detection limit was 17.3 pg/mL. The assay range was 0.14–8.83 nM.

## Conflicts of Interest

The authors declare no conflicts of interest.

## Supporting information

Supplementary Material

## Data Availability

The data that support the findings of this study are available from the corresponding author upon reasonable request.
