# Supplementary material for: Selective and Fast Cortisol Sensing in Human Saliva Using Graphene‐Based On‐Chip Field‐Effect Transistors
Source: Small Sci. 2026 Sep 27;6(10):e70405. doi: 10.1002/smsc.70405 (PMC13618521; doi:10.1002/smsc.70405)
Supplement: Supplementary file 1 [file SMSC-6-e70405-s001.pdf]

Supporting Information

Selective and Fast Cortisol Sensing in Human Saliva Using Graphene-based On-Chip Field-Effect Transistors

Bajramshahe Shkodra, Ksenija Arslanova, Eva Fellinger, Tanja Stimpel-Lindner, Sebastian Klenk, Sebastian Hauk, Kathrin C. Knirsch, Andreas Hirsch, Kristiina Iljin, Tarja K. Nevanen, Annette Schmidt, Georg S. Duesberg*

[1] B. Shkodra, K. Arslanova, T. Stimpel-Lindner, S. Klenk, G. S. Duesberg
Institute of Physics & Research Center SENS, Faculty of Electric Power Systems and Information Technology, University of the Bundeswehr Munich, Werner-Heisenberg-Weg 39, 85579 Neubiberg, Germany Email Address: bajramshahe.shkodra@unibw.de; duesberg@unibw.de
[2] E. Fellinger, A. Schmidt
Institute for Sports Science, Faculty of Human Sciences, University of the Bundeswehr Munich, Werner-Heisenberg-Weg 39, 85579 Neubiberg, Germany
[3] S. Hauk, K. C. Knirsch, A. Hirsch
Department of Chemistry and Pharmacy, Friedrich-Alexander University of Erlangen-Nuremberg (FAU), Nikolaus-Fiebiger-Straße 10, 91058 Erlangen, Germany
[4] K. Iljin, T. K. Nevanen
VTT Technical Research Centre of Finland, Tekniikantie 21, 02150 Espoo (Finland)

[a] Authors to whom correspondence should be addressed:
bajramshahe.shkodra@unibw.de, duesberg@unibw.de

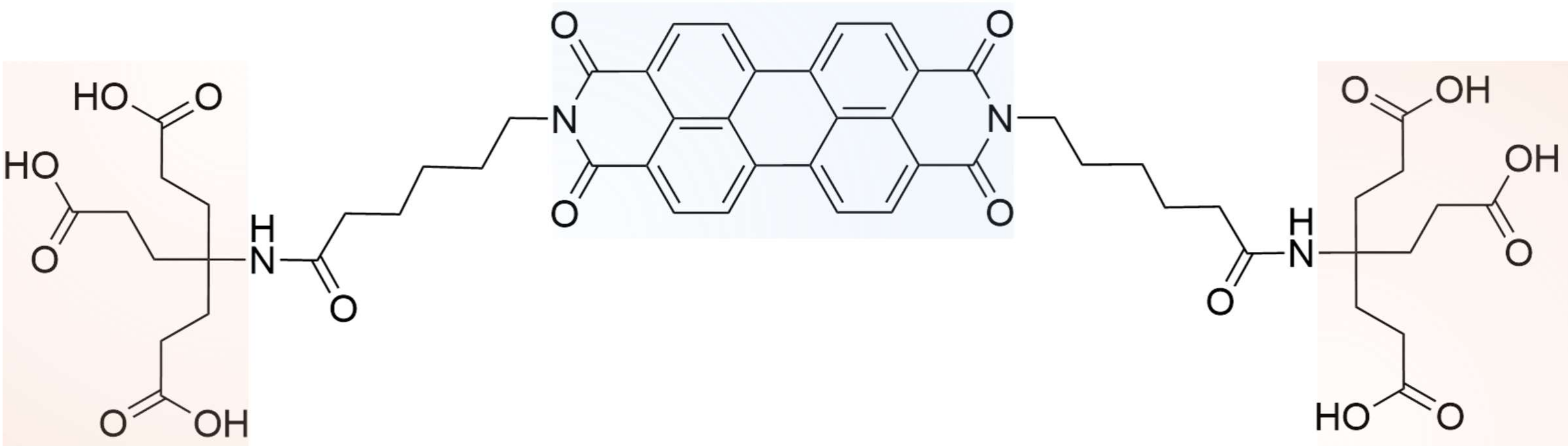

Figure S1: Perylene bisimide (PBI) molecular structure. The planar aromatic core region is highlighted in blue, the carboxylated groups are highlighted in orange.

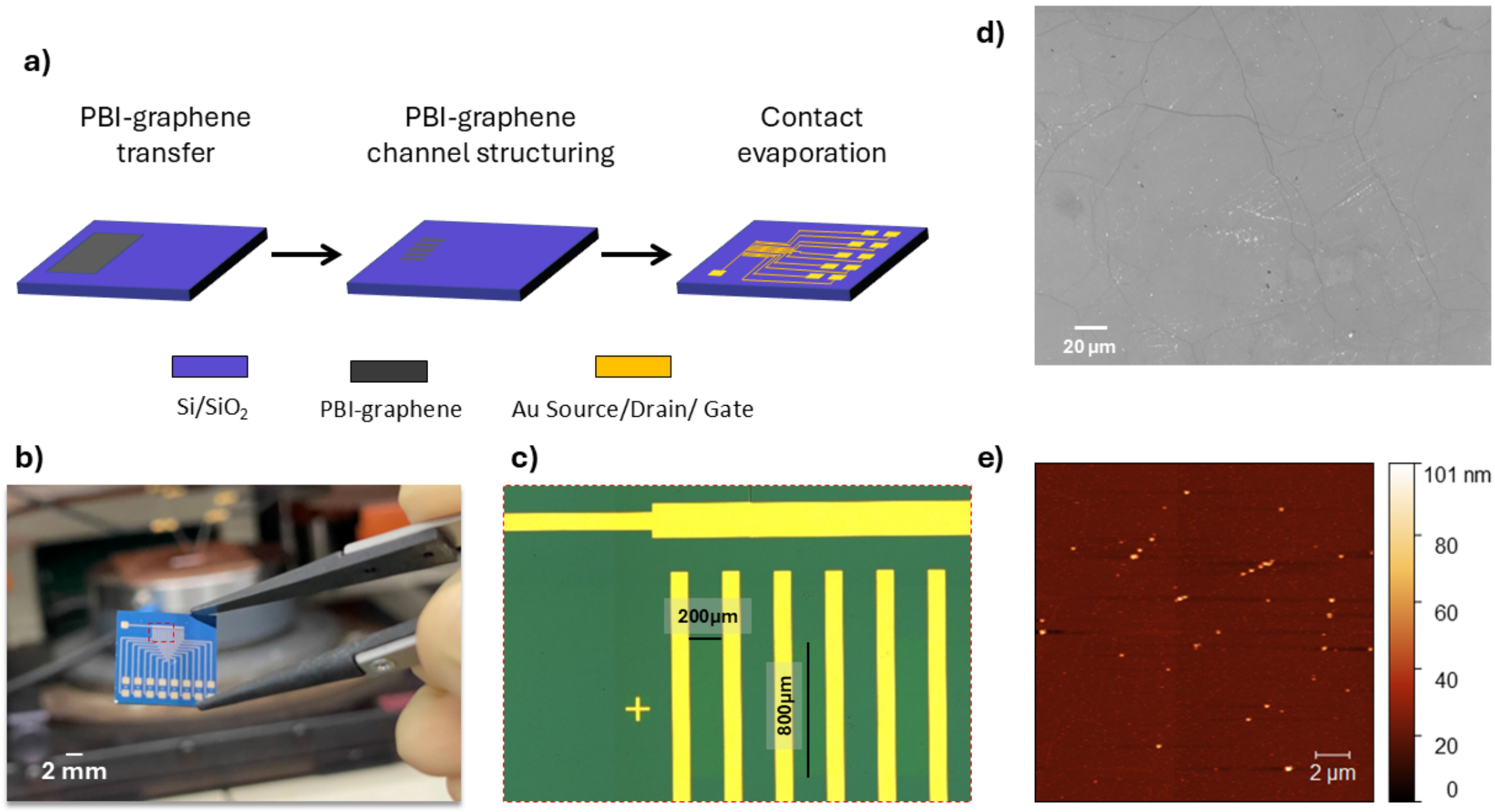

Figure S2: a)Following the PBI functionalization, the Cu-graphene foil was spin-coated with poly(methyl methacrylate) (PMMA, 200K A4, Allresist GmbH) and baked at 110 °C for 10 min. The graphene on the backside of the Cu foil was then selectively removed by treatment with a 1:1 mixture of H_2O_2 and H_2SO_4, followed by thorough rinsing with deionized water. Subsequently, the copper substrate was etched in a 0.2 M ammonium persulfate (APS) solution for 20 h, and the PBI-graphene/PMMA stack was transferred onto a Si/SiO_2 substrate. After drying overnight in a desiccator, the PMMA layer was removed by immersion in acetone (two cycles of 15 min each). Device fabrication involved two successive photolithography steps. The first step defined the graphene channels with dimensions of 200 µm length and 800 µm width. After patterning, unwanted PBI-graphene outside the channel regions was removed by oxygen plasma etching. The second lithography step defined the source, drain, and gate electrodes on the same substrate. Metal contacts consisting of a 10 nm nickel adhesion layer and 50 nm gold were deposited *via* thermal evaporation (MBRAUN ProVap 5G system with INFICON SQM-160 rate/thickness monitor), followed by lift-off. To remove residual polymers, the devices were immersed in boiling acetone for 6 h and subsequently annealed under vacuum (1×10^{-4} mbar) at 250 °C for 20 h. b) Picture of as fabricated GFET. c) Microscope image of the fabricated GFET with a zoom in channel area. d) SEM image of the GFET channel. e) AFM micrograph of the GFET channel.

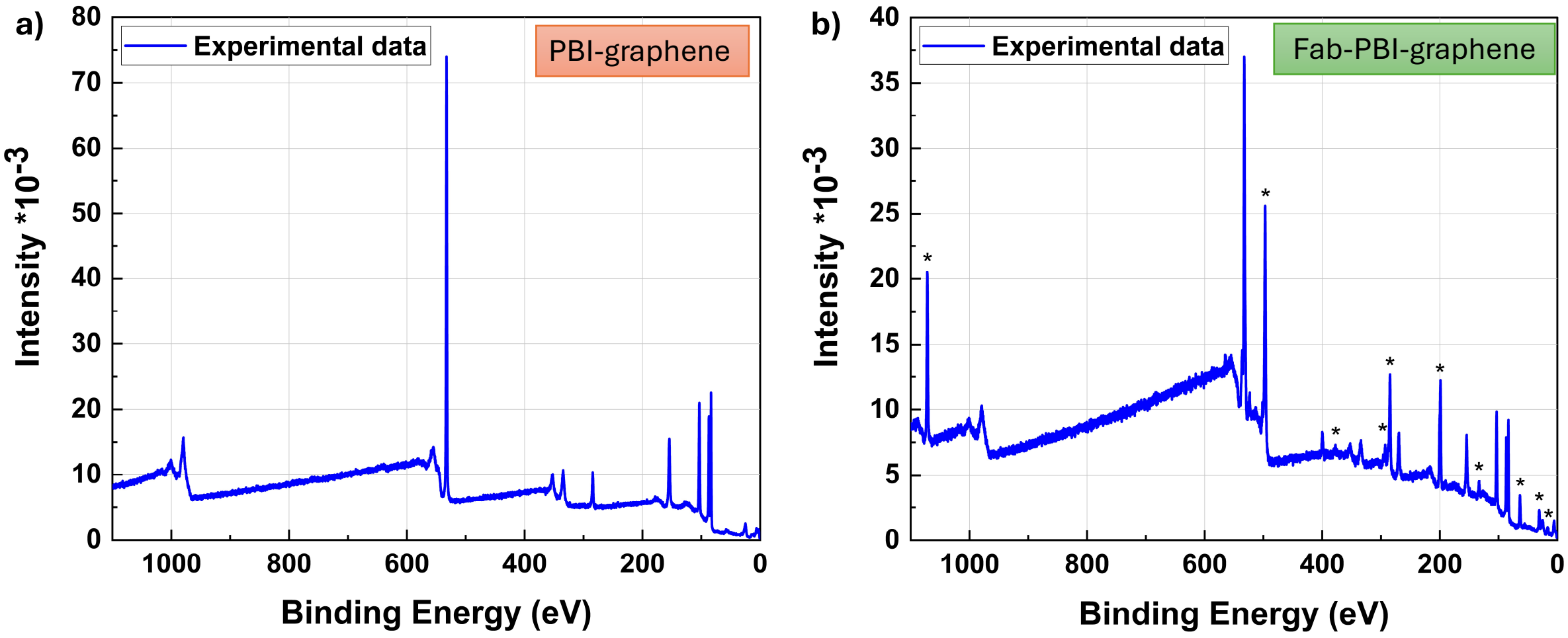

Figure S3: XPS survey spectra of a) PBI-graphene b) Fab-PBI-graphene.Peaks arising from impurities (Na, Cl, K) are marked with asterisks. These can be attributed to buffer residues on the surface.

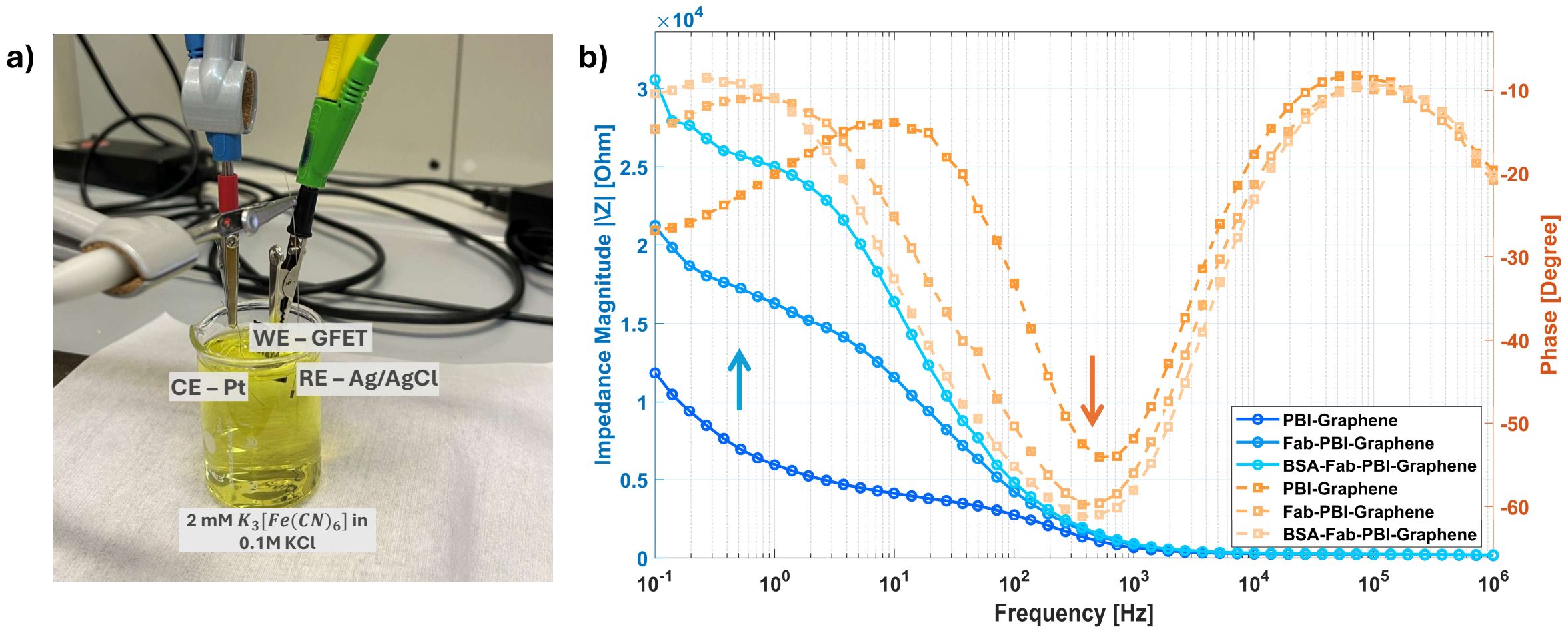

Figure S4: a) The electrochemical measurements setup: the PBI-graphene channel served as the working electrode - WE (with source and drain electrodes short-circuited), a platinum wire as the counter electrode - CE, and a Ag/AgCl electrode as the reference electrode - RE. Measurements were perform in 2 mM ferricyanide ($[Fe(CN)_6]^{3-}$) redox reporter in 0.1 M KCl. b) The Bode plots: impedance (blue lines) and phase angle (orange lines) were recorded from 10^2 to 10×10^5 Hz to describe the EG-GFET surface modification. The impedance distinctly increases after modification with Fab fragments followed by BSA blocking. This increase is attributed to the thicker dielectric layer that is formed on the PBI-graphene surface due to the combined Fab-fragment and BSA functionalization, which reduces the conductivity and thus increases the impedance. The observed lag in phase angle after functionalization indicates changes in the interfacial capacitance. More capacitive behavior is evident at mid frequencies for the functionalized channel, where the phase angle reaches $-60.95°$ compared to $54.06°$ for the bare PBI-graphene channel, demonstrating successful functionalization.

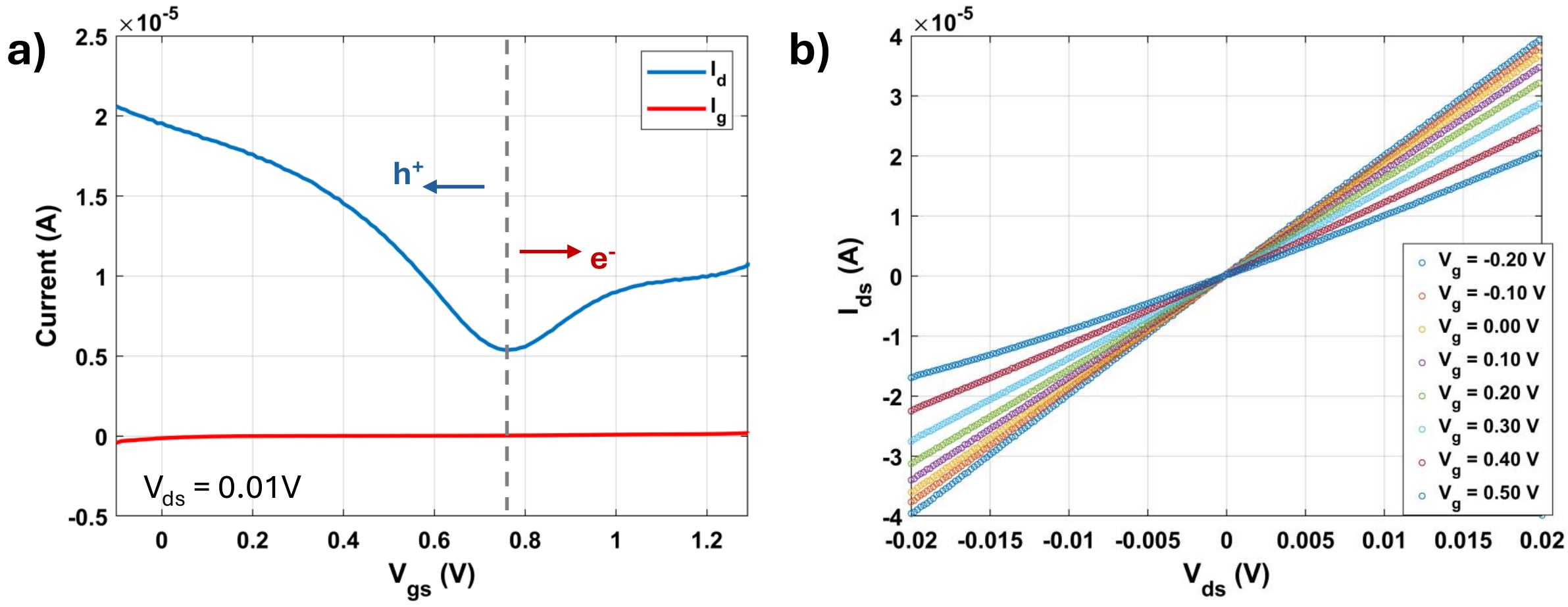

Figure S5: Electrical characterization of electrolyte-gated graphene field-effect transistor (EG-GFET) biosensor operated with a liquid side gate. a) The transfer characteristic shows a typical ambipolar behavior (blue curve) of the fully biofunctionalized EG-GFET device measured in 1× PBS, with a V_{Dirac} of 0.76 V. The gate current remained at least two orders of magnitude lower than the drain current during the entire sweep (red curve). b) The output characteristics were recorded by varying the V_{ds} from −0.02 V to 0.02 V for different values of V_{gs} raging from −0.2 V to 0.5 V, in 0.1 V steps. The linear relationship between I_{ds} and V_{ds} confirms ohmic contact between graphene and the metal electrodes.

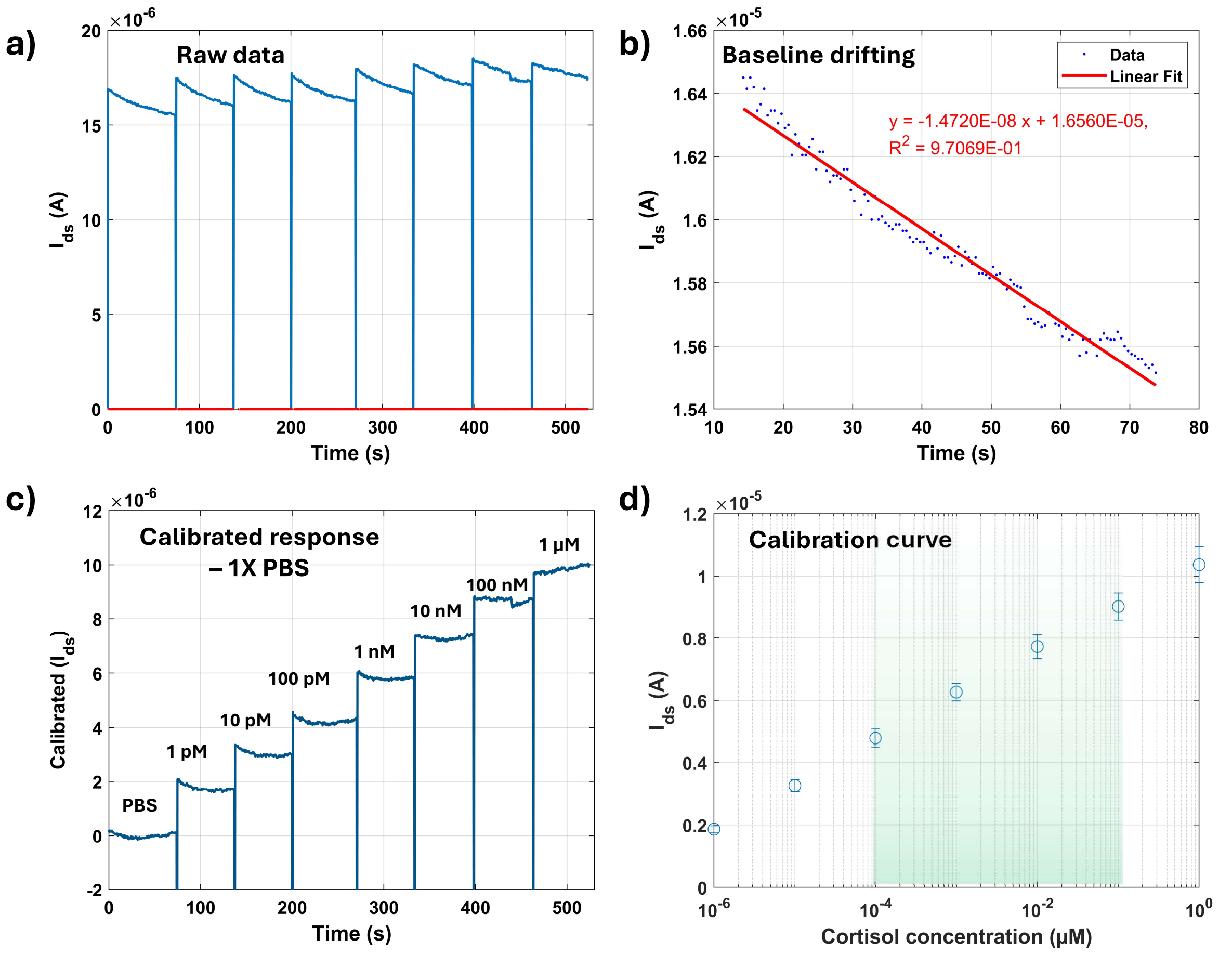

Figure S6: a) Representative real-time measurement of the drain–source current (blue line) and the gate current (red line), for fixed $V_{ds} = 0.01\,V$ and $V_{gs} = 0.4\,V$. b) The baseline I_{ds} showed a linear decrease (*i.e.,* for this specific device, a decrease of 14.7 nA/s with a coefficient of determination of 97.07 %). It was therefore possible to subtract this linear trend from the response of the devices to different concentrations of cortisol (calibrated response). c) Calibrated I_{ds} (*i.e.,* after the baseline subtraction), used for the extraction of the calibration curve. d) The calibration curve was determined by averaging the final 30 s of the I_{ds} for each cortisol concentration.

Table S1: Composition of 1x PBS and Artificial Sweat

	**1x PBS**	**Artificial Sweat**
**Component**	**Concentration (mM)**	**Concentration (mM)**
NaCl	137	30
KCl	2.7	8
Na_2HPO_4	10.1	–
KH_2PO_4	1.8	–
Creatinine	–	0.084
Glucose	–	0.17
Uric acid	–	0.059
Ascorbic acid	–	0.01

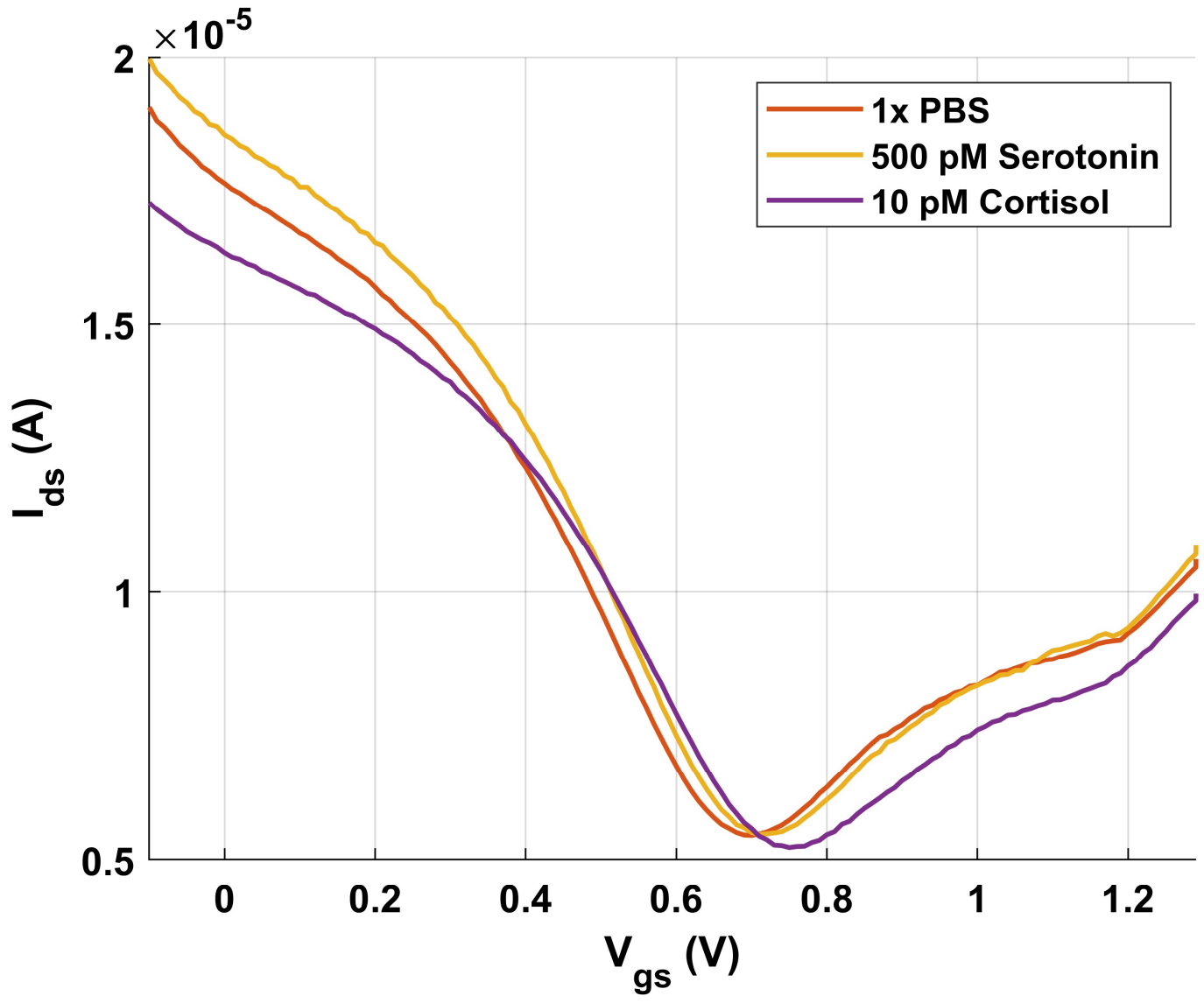

Figure S7: Transfer curve of the EG-GFET showing minimal response to 500 pM serotonin, while the addition of 10 pM cortisol produces a clear shift toward higher V_{gs}, confirming selective cortisol recognition.

Table S2: Comparison of recent carbon nanomaterial-based EG-FET cortisol biosensors

**Channel materials**	**Biorecognition element**	**Electrolyte**	**Detection range**	**LOD (fM)**	**Reference**
CNT	Aptamer	0.01x PBS	1 fM to 1 µM	1	(1)
CNT	Antibody	Artificial sweat	1 pM to 1 µM	220	(2)
CNT	Aptamer	1x PBS	1 nM to 1000 nM	2.7×10^6	(3)
Graphene	Aptamer	1x PBS	1 pM to 1 µM	1000	(4)
Graphene	Antibody	Artificial tear	2.7 nM to 110 nM	2.7×10^4	(5)
Graphene	Antibody	Artificial sweat	500 fM to 100 nM	500	(6)
Graphene	Antibody fragments	1xPBS, Artificial sweat, Human saliva	100 fM to 1 µM	30	This work

References

[1] T. T. H. Nguyen, C. M. Nguyen, M. A. Huynh, Q. T. Trinh, P. Tanner, S. Ingebrandt, X. T. Vu, T.-K. Nguyen, and N.-T. Nguyen, "Ultra-sensitive aptameric field-effect transistor for cortisol sensing," *Sensors and Actuators Reports*, vol. 9, p. 100324, 2025.

[2] L. Bian, W. Shao, Z. Liu, Z. Zeng, and A. Star, "Detection of stress hormone with semiconducting single-walled carbon nanotube-based field-effect transistors," *Journal of The Electrochemical Society*, vol. 169, no. 5, p. 057519, 2022.

[3] B. Mirka, J. Ding, and F. Lapointe, "Tight-anchoring of gold nanoparticles to polymer-wrapped semiconducting single-walled carbon nanotubes for biosensor applications," *ACS Applied Nano Materials*, vol. 8, no. 36, pp. 17 397–17 408, 2025.

[4] A. Gilani, A. Saeidi, S. Sheibani, J. Longo, S. Kamaei, A. Ameti, N. Niederländer, N. Pitteloud, and A. M. Ionescu, "Aptamer-decorated graphene channel array with liquid-gating for sensing cortisol stress hormone," in *2024 IEEE BioSensors Conference (BioSensors)*. IEEE, 2024, pp. 01–04.

[5] M. Ku, J. Kim, J.-E. Won, W. Kang, Y.-G. Park, J. Park, J.-H. Lee, J. Cheon, H. H. Lee, and J.-U. Park, "Smart, soft contact lens for wireless immunosensing of cortisol," *Science advances*, vol. 6, no. 28, p. eabb2891, 2020.

[6] K. H. Kim, J.-T. Kim, S. E. Seo, H. G. Lee, and O. S. Kwon, "Immuno-graphene field-effect transistor-based cortisol bioelectronics using a novel cortisol antibody," *BioChip Journal*, vol. 19, no. 2, pp. 194–203, 2025.
